# Pleiotrophin as a central nervous system neuromodulator, evidences from the hippocampus

**DOI:** 10.3389/fncel.2014.00443

**Published:** 2015-01-08

**Authors:** Celia González-Castillo, Daniel Ortuño-Sahagún, Carolina Guzmán-Brambila, Mercè Pallàs, Argelia Esperanza Rojas-Mayorquín

**Affiliations:** ^1^Doctorwado en Ciencias en Biología Molecular en Medicina (DCBMM), CUCS, Universidad de GuadalajaraGuadalajara, Jalisco, México; ^2^Instituto de Investigación en Ciencias Biomédicas (IICB), CUCS, Universidad de Guadalajara, GuadalajaraJalisco, México; ^3^Tecnológico de Monterrey, División de Biotecnología y Salud, Escuela de Medicina, Campus GuadalajaraGuadalajara, Jalisco, México; ^4^Department of Pharmacology and Medical Chemistry, Faculty of Pharmacy School of Pharmacy, Institute of Biomedicine (IBUB), Centros de Investigación Biomédica en Red de Enfermedades Neurodegenerativas (CIBERNED), University of BarcelonaBarcelona, Spain; ^5^Departamento de Ciencias Ambientales, Instituto de Neurociencias, CUCBA, Universidad de GuadalajaraGuadalajara, Jalisco, México

**Keywords:** pleiotrophin, neuromodulation, hippocampus, neuropeptide, miple

## Abstract

Pleiotrophin (PTN) is a secreted growth factor, and also a cytokine, associated with the extracellular matrix, which has recently starting to attract attention as a significant neuromodulator with multiple neuronal functions during development. PTN is expressed in several tissues, where its signals are generally related with cell proliferation, growth, and differentiation by acting through different receptors. In Central Nervous System (CNS), PTN exerts post-developmental neurotrophic and -protective effects, and additionally has been involved in neurodegenerative diseases and neural disorders. Studies in* Drosophila* shed light on some aspects of the different levels of regulatory control of PTN invertebrate homologs. Specifically in hippocampus, recent evidence from PTN Knock-out (KO) mice involves PTN functioning in learning and memory. In this paper, we summarize, discuss, and contrast the most recent advances and results that lead to proposing a PTN as a neuromodulatory molecule in the CNS, particularly in hippocampus.

## Introduction

Pleiotrophin (PTN) is a secreted cell signaling cytokine that acts as growth factor associated with the extracellular matrix, which has recently started to come to the fore as a significant neuromodulator with multiple neuronal functions. PTN is an 18-KDa protein and has 168 amino acids. It was discovered practically simultaneously by several laboratories nearly 25 years ago; thus, it initially received several names as follows: HBGF-8 (Heparin-binding growth factor; Milner et al., 1989); HB-GAM (Heparin-binding growth-associated molecule; Rauvala, 1989; Merenmies and Rauvala, 1990); HBNF (Heparin-binding neutrophil factor; Kovesdi et al., 1990); OSF-1 (Osteoblast-specific factor 1; Tezuka et al., 1990), and HARP (Heparin affinity regulatory peptide; Courty et al., 1991).

PTN shares high homology (>50%) with another peptide, denominated Midkine (MK); both are highly conserved throughout evolution and are found in species ranging from *Drosophila* to humans (Kadomatsu and Muramatsu, 2004). This means that although both have many functions in common and participate in similar functions, they also possess more particular, specific, and non-redundant functions. It is evident when both are simultaneously knocked out in mice, they display severe abnormality phenotypes. However, when independently knocked out, PTN−/− and MDK−/− mice are far from being completely normal and exhibit moderate but different abnormalities (Muramatsu et al., 2006; Zou et al., 2006; Gramage and Herradón, 2010; Himburg et al., 2012; Vicente-Rodríguez et al., 2013), which denotes that although both peptides could present overlapping or similar functions, they are also clearly involved in different roles.

## PTN could signal through a multi-receptor complex

**Figure 1:**
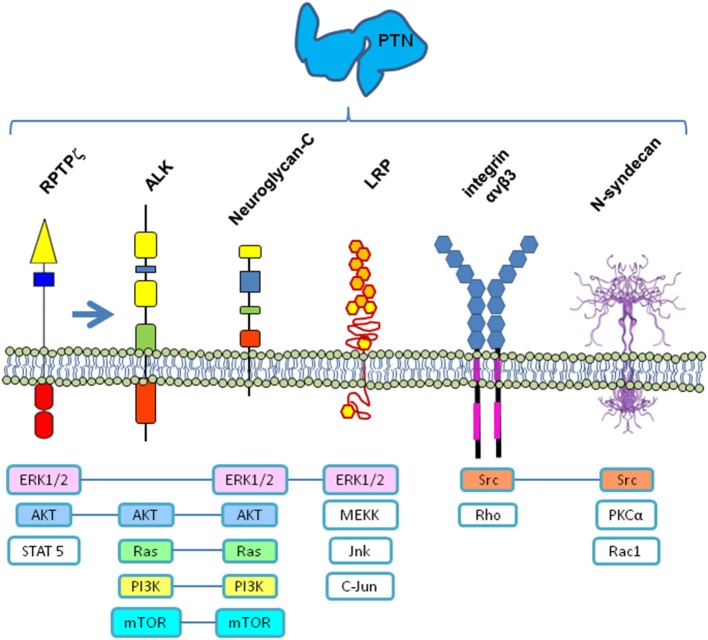
**Receptors and signaling pathways possibly involved in PTN signaling**. All or some of these membrane receptors could function as a multi-molecular complex coordinated to transduce the PTN signal into the cell by different signaling pathways. **RPTPζ**—Receptor protein tyrosine phosphatase ζ, EC = 3.1.3.48); **ALK**—Anaplastic Lymphoma Kinase; **LRP**—Low-density lipoprotein receptor-related protein; **ERK1/2**—Extracellular-Signal-Regulated Kinase; **AKT**—Serine/Threonine-specific protein kinase; **STAT5**—Signal transducer and activator of transcription 5; **Ras**—Rat sarcoma small GTP-ase; **PI3K**—Phosphatidylinositol-4,5-bisphosphate 3-kinase; **mTOR**—Mechanistic target of Rapamycin (serine/threonine kinase); **MEKK**—mitogen-activated protein Kinase/ERK kinase kinase 3; **Jnk**—c-Jun N-terminal kinase; **Src**—Sarcoma tyrosin kinase; **Rho**—Ras homology small GTPase; **PKCα**—Protein kinase C alpha; **Rac1**—Ras related small GTPase. N-syndecan structure from www.ebi.ac.uk

PTN signals are generally related with cell proliferation, growth and differentiation, but PTN has also has been involved in other functions by acting through different receptors (Figure 1). Mainly, PTN can bind and signal via Receptor protein tyrosine phosphatase ζ (RPTPζ), EC = 3.1.3.48 (Maeda et al., 1996, 1999; Meng et al., 2000), which is a transmembrane chondroitin sulfate proteoglycan present in two isoforms (shorter and full-length), which in turn also binds with various cell adhesion molecules (NrCAM, L1/Ng-CAM, contactin, N-CAM, and TAG1), growth factors (PTN, MK, and fibroblast growth factor (FGF-2), and extracellular matrix molecules (amphoterin, tenascin-C, and tenascin-R) (reviewed in Maeda et al., 2010). Under certain circumstances, PTN can act via Anaplastic Lymphoma Kinase (ALK) receptor (Stoica et al., 2001, 2002; Powers et al., 2002), although some evidences suggest that the action of PTN on ALK could occur through its previous interaction with RPTPζ (Perez-Pinera et al., 2007). Additionally, PTN; (1) promote neurite outgrowth via N-syndecan receptor (Raulo et al., 1994) or via Neuroglycan-C (NGC; Nakanishi et al., 2010), (2) interact with integrin ανβ3 (alpha nu beta 3) receptor, which is a mechano-sensitive cell membrane receptor, for cell adhesion (Mikelis et al., 2009), and (3) interact with Low-density lipoprotein (LDL) Receptor-related protein (LRP; Kadomatsu and Muramatsu, 2004). Additionally, two different species of PTN, PTN15 and PTN18, have been described (Lu et al., 2005), but their differential interaction or their affinities to different receptors has not yet been established, which adds another level of complexity to their physiological functioning.

It has been recently proposed that PTN signaling may function through a multi-receptor complex (Xu et al., 2014), combining the previously mentioned receptors, and most probably other adaptor proteins, which interact under certain circumstances inside particular cell membrane microdomains, probably also associated with lipids in raft configuration, which could explain the variety of functions in different tissues, in terms of the combinatorial analysis of the elements present at each time and place. Then, PTN action over previously mentioned receptors could in turn signal through different signal pathways (Figure 1). Increasing our knowledge of the intricate molecular mechanisms involved would clarify the receptor complexes and signaling pathways implicated, as well as advance the discovery of other molecules involved, which in turn will lead us to fully explain its variety of functions.

## Differential expression of PTN receptors during development and in adult could indicate its dissimilar participation in different functions

Although during early development PTN expression is widely distributed in Central Nervous System (CNS; Li et al., 1990), expression of PTN in adult brain appears to be constitutive and apparently limited to only a few cell types in brain cortex, hippocampus, cerebellum and olfactory bulb (Wanaka et al., 1993; Lauri et al., 1996; Basille-Dugay et al., 2013), as well as in some striatal interneurons (Taravini et al., 2005). At these locations, the differential expression of its receptors could exist, which might partially explain its diverse actions. RPTPζ is expressed in glial cells as well as neurons. In hippocampal cells, it is located at the postsynaptic membrane of pyramidal neurons in adult (Hayashi et al., 2005), and its expression is modulated by spatial learning (Robles et al., 2003); therefore, it is involved in learning and long-term memory. Additionally, it is highly expressed following injury in areas of axonal sprouting and glial scarring (Snyder et al., 1996), and its expression is induced in inner molecular-layer astrocytes of the dentate gyrus of the sclerotic hippocampus in patients with epilepsy (Perosa et al., 2002). Also, it is involved in regulating dendritogenesis and synaptogenesis of hippocampal neurons *in vitro* (Asai et al., 2009). Therefore, PTN signaling through RPTPζ could be involved in modulation of hippocampal plasticity during learning and also during recovery after a lesion or in neuropathological situations, by modulating dendritogenesis and synaptogenesis. RPTP-ζ/β might be implicated in plastic rearrangements of nigrostriatal connections, such as sprouting of dopaminergic terminals or postsynaptic changes triggered by L-DOPA treatment in a model of Parkinson disease (Ferrario et al., 2008).

Likewise, ALK receptor is expressed in adult mammalian hippocampus and has also been implicated in neurogenesis, memory, and learning (Weiss et al., 2012). In addition, it has been involved in basal hippocampal progenitor proliferation and its deficiency induces alterations in behavioral tests (Bilsland et al., 2008). Although MK has been postulated to be the ligand for ALK receptor, at least in controlling sympathetic neurogenesis (Reiff et al., 2011), PTN also appears to be able to interact with this receptor (Stoica et al., 2001), although this remains controversial (Mathivet et al., 2007), and it appears that PTN performs its action on ALK thought its previous interaction with RPTPζ (Perez-Pinera et al., 2007).

It is clear that overlapping of PTN and MK activities can occur in some cases, but certainly not under all circumstances, as mistakenly suggested by Xu et al. (see Figure 3 in Xu et al., 2014). Although both peptides exhibit similar actions under certain physiological conditions, at least in the CNS, each also exerts diverse effects and performs different actions, depending on the cerebral region, as mentioned later.

## Studies in *Drosophila* enlighten some aspects of the different levels of regulatory control of PTN expression and function in their invertebrate homologs

*Drosophila* homologs to MK and PTN are Miple1 and Miple2, with 20 and 24% identical to human MK and human PTN, respectively (Englund et al., 2006). However, they cannot be assigned as respective homologs, but only as members of the same family. Respective genes are arranged in tandem, suggesting that they have arisen as a result of a gene duplication event at some point of evolution. However, these secreted proteins are expressed in restricted, non-overlapping patterns, with Miple1 mainly expressed in the developing embryonic nervous system, while miple2 is strongly expressed in the developing gut endoderm (Englund et al., 2006). Therefore, had they been generated by gene duplication, they were clearly submitted to different selective pressure expression regulation, and consequently diverge in their expression pattern, and most probably in functioning. Thus, it will be relevant to elucidate, in useful model such as *Drosophila*, the molecular interactions of these peptides during complex developmental processes.

The messenger RNA (mRNA) 3′-Untranslated region (UTR) binding protein HOW (Held out wing) is able to post-translationally repress *miple*, downregulating its mRNA levels in mesoderm in order to enable proper mesoderm spreading during early embryogenesis in *Drosophila* (Toledano-Katchalski et al., 2007). This suggests that a similar mechanism could drive some regulatory action over PTN and MK expression in vertebrates.

Another point of regulation corresponds to the interaction of miple, as a signaling peptide, with other proteins. For example, by affecting the affinity of HTL ligands to the HTL receptor (Heartless, a *Drosophila* FGF receptor), thereby modulating the strength of HTL-dependent signaling (Toledano-Katchalski et al., 2007). Thus, it is feasible that PTN could interact with other peptides being a key modulator in the binding process to different complexes of receptors.

Interestingly, the combined expression pattern of Miple1 and Miple2 complements the expression pattern of the *Drosophila* ALK homolog (DAlk; Lorén et al., 2001, 2003). However, its ligand has been identified as a different peptide, namely Jelly belly (Jeb), which play roles in neuromuscular junction growth and function, early mesoderm development, and also in axon targeting of photoreceptors (Weiss et al., 2001; Englund et al., 2003; Lee et al., 2003; Bazigou et al., 2007; Rohrbough and Broadie, 2010). It is relevant to mention that *Drosophila* Jeb is not able to activate mouse ALK (Yang et al., 2007), and Jeb homologs in vertebrates have not yet been described. However, it is noteworthy that secreted Jeb contains a LDL receptor class A domain that contains 6 disulphide-bound cysteines (Bieri et al., 1995), and could constitute a binding site for LDL and calcium (Yamamoto et al., 1984). Given that LRP is a LDL receptor-related protein involved in PTN action in vertebrates (Kadomatsu and Muramatsu, 2004), it would be possible that Jeb signaling could be related with miple signaling and their vertebrate counterpart is unveiled to date.

Based on all previous cited evidences, and given the complexity of the molecular interactions in which PTN is clearly involved, it will be necessary to widely divulge approaches for disclosing its functioning. In this respect, one of the most useful approaches could be analysis by microarrays of the gene profile expression in PTN-defective Knock-out (KO) mice. Recently, in our laboratory, we performed these experiments and established the differential gene expression in the hippocampus of PTN KO mice (In preparation).

## Differential effects of PTN vs. MDK indicate it as a neuromodulatory peptide in CNS, particularly in hippocampus

PTN and MK have been shown to induce and stimulate neuronal differentiation (Jung et al., 2004; Ishikawa et al., 2009; Luo et al., 2012). More specifically, PTN has been involved in lineage-specific differentiation of glial progenitor cells, axonal outgrowth, synaptic plasticity, and angiogenesis (Mitsiadis et al., 1995; Kadomatsu and Muramatsu, 2004). PTN participates in axon regeneration after injury, being highly expressed by reactive astrocytes (Iseki et al., 2002) as a source of trophic support for neurons in brain (Dugas et al., 2008) and rescuing nigral dopaminergic neurons from degeneration (Hida et al., 2007; Moses et al., 2008). However, its precise molecular mechanisms remain unknown.

In addition to these widely recognized roles of PTN, functionally, PTN−/− mice exhibited a delayed response to nociceptive stimulus in the tail-flick test (Gramage and Herradón, 2010), and clonidine-induced analgesia was significantly enhanced (Vicente-Rodríguez et al., 2013) when compared with MK−/− and Wild-type (WT+/+) mice. These evidences strongly suggest that endogenous PTN modulates nociceptive transmission at the spinal level.

In addition, PTN has been involved in neurodegenerative disorders and in response to chronic drug consumption. PTN is upregulated in cortex and caudate-putamen after injection of a cannabinol (Mailleux et al., 1994), and in nucleus accumbens after acute administration of amphetamine (Le Grevès, 2005); in addition, it is also highly upregulated in substantia nigra of patients with Parkinson disease (Marchionini et al., 2007) and treatment with L-Dopa increases PTN levels in striatum (Ferrario et al., 2004). Thus, it has been involved, as is MK (Prediger et al., 2011), in regulation of the survival and function of dopaminergic neurons (Jung et al., 2004). Taken together, this evidence supports the hypothesis that PTN is upregulated in neurodegenerative and addictive disorders in order to induce trophic or neuroprotective effects on dopaminergic neurons (Herradón and Pérez-García, 2014).

After PTN expression was described in hippocampus (Bloch et al., 1992; Vanderwinden et al., 1992; Wanaka et al., 1993), it was suggested that it plays a role in injury-induced response (Takeda et al., 1995; Poulsen et al., 2000) and activity-dependent plasticity (Lauri et al., 1996; Rauvala and Peng, 1997) in rat hippocampus, by affecting early, synapse-specific stages of LTP production (Lauri et al., 1998). Later, it was demonstrated, in PTN-deficient mice, that hippocampal slices exhibit a lowered threshold for induction of LTP (Amet et al., 2001) and that LTP was attenuated in mice overexpressing PTN (Pavlov et al., 2002), possibly by enhancing GABAergic inhibition in CA1 (Pavlov et al., 2006) and affecting recognition memory (del Olmo et al., 2009). Together, these evidences indicate that PTN could act as inducible signal to inhibit LTP in the hippocampus. Therefore, taken collectively, these evidences add a new role to the previous functions referred for PTN, thus functioning as a neuromodulatory factor in the hippocampus (Table 1). However, molecular evidence continues to be incomplete regarding the complex signaling system involved in PTN modulation.

**Table 1:** **PTN functions**.

Classical functions	REFs
Growth factor
Cell growth, cell proliferation, cell differentiation	Maeda et al. (1996, 1999); Meng et al. (2000)
Cell adhesion
**Functions as Neuromodulatory molecule in CNS**
Neurogenesis and neural migration and differentiation
Axonal outgrowth	Mitsiadis et al. (1995); Kadomatsu and Muramatsu (2004)
Dendritogenesis and synaptogenesis	Asai et al. (2009)
Learning and long-term memory
Modulating LTP by activity-dependent plasticity	Lauri et al. (1996); Rauvala and Peng (1997)
Neuritogenesis and neurite extension	Bao et al. (2005); Raulo et al. (2005); Nakanishi et al. (2006)
Dendritogenesis and synaptogenesis	Asai et al. (2009)
Modulates nociceptive transmission
Neuroprotective effects
Injury-induced response	Takeda et al. (1995); Poulsen et al. (2000)
Regeneration after injury	Iseki et al. (2002)
Involved in neurodegenerative disorders	Mailleux et al. (1994)
Response to chronic drug consumption	Mailleux et al. (1994)

*PTN, Pleiotrophin; CNS, Central nervous system*.

To complete a whole view and to fully understand the modulatory role of PTN in CNS, and particularly in hippocampus, it is necessary first to establish which elements of the molecular machinery are present, and second, which are the ways in which they interact with each other. In this respect, immunohistochemical analyses reveal that RPTPζ and its substrate, GIT1/Cat-1, are co-localized in the processes of pyramidal cells in hippocampus and neocortex in rat brain, and PTN increases tyrosine phosphorylation of GIT1/Cat-1 in neuroblastoma B103 cells (Kawachi et al., 2001). Also, PSD-95/SAP90 family proteins, along with RPTPζ, are distributed in the dendrites of pyramidal neurons of hippocampus and neocortex (Kawachi et al., 1999). Additionally, it has been demonstrated that P190 RhoGAP activity, regulated by PTN/RPTPζ pathway, is involved in hippocampus-dependent memory formation through the downstream Rho/Rock pathway, which plays an important role in cell migration, axonal growth, and synaptic plasticity (Tamura et al., 2006). Another receptor involved in PTN signaling is N-syndecan receptor (Raulo et al., 1994), which due to deficiency in hippocampus exhibits enhanced LTP and altered hippocampus-dependent memory (Kaksonen et al., 2002). Moreover, this KO mouse is not responsive to PTN.

On the other hand, PTN regulates neurite extension and plasticity in pig hippocampal neurons *in vitro*, signaling through chondroitin sulfate/dermatan sulfate hybrid chains (Bao et al., 2005; Raulo et al., 2005); this action could involve chondroitin sulfate E as a binding partner, co-receptor, or genuine receptor for PTN (Deepa et al., 2002), but it is also reasonable to speculate that this could involve NGC, a brain-specific chondroitin sulfate proteoglycan involved in neuritogenesis (Nakanishi et al., 2006) and which interacts with PTN (Nakanishi et al., 2010).

## Concluding remarks

Therefore, the different actions of PTN as a neuromodulatory peptide (Table 1) could vary during development depending on the signaling pathways that it mainly activates. During early brain development, PTN implication in regulating neurogenesis and neural migration and differentiation, regulating axonal outgrowth, dendritogenesis, and synaptogenesis, could principally involve signaling through PTN/RPTPζ, and also through integrin αν β3, possibly acting coordinately. Later, in adult, the participation of PTN in learning and long-term memory, by modulating LTP by activity-dependent plasticity memory process in hippocampus, can again be principally mediated by its signaling through PTN/RPTPζ, possibly in combination with its signaling through N-syndecan pathway. Finally, its neuroprotective effects constitute a relevant role, suggesting that PTN signaling pathways are involved in neurodegenerative disorders, as well as in response to injuries and chronic drug consumption. Those signaling pathways may be functioning through a multi-molecular complex of receptors, combining previously mentioned receptors and other adaptor proteins, which interact inside membrane microdomains in raft configuration, which could explain each of these functions.

We are sure that a lot of molecules involved in PTN signaling pathways remain unknown to date. It is necessary to perform more integral studies, such as the use of proteomics and genomics approaches, as well as studies *in vivo* (employing PTN-KO) and *in vitro* (by mean of experiments with small interfering RNA [siRNA]), which will undoubtedly elucidate the complete molecular mechanisms involved.

## Author statement

Daniel Ortuño-Sahagún, Argelia E. Rojas-Mayorquín, Mercè Pallàs Conceived the work, drafted and revised critically.

Celia González-Castillo, Carolina Guzmán-Brambila, Acquire and compilates information.

All authors, Write and revised the manuscript.

## Conflict of interest statement

The authors declare that the research was conducted in the absence of any commercial or financial relationships that could be construed as a potential conflict of interest.
